# Mesoscale brain explorer, a flexible python-based image analysis and visualization tool

**DOI:** 10.1117/1.NPh.4.3.031210

**Published:** 2017-05-19

**Authors:** Dirk Haupt, Matthieu P. Vanni, Federico Bolanos, Catalin Mitelut, Jeffrey M. LeDue, Tim H. Murphy

**Affiliations:** a University of British Columbia, Kinsmen Laboratory of Neurological Research, Faculty of Medicine, Department of Psychiatry, Vancouver, Canada; b University of British Columbia, Djavad Mowafaghian Centre for Brain Health, Vancouver, Canada

**Keywords:** connectome, optogenetics, cortex, brain imaging, mesoscale, widefield

## Abstract

Imaging of mesoscale brain activity is used to map interactions between brain regions. This work has benefited from the pioneering studies of Grinvald et al., who employed optical methods to image brain function by exploiting the properties of intrinsic optical signals and small molecule voltage-sensitive dyes. Mesoscale interareal brain imaging techniques have been advanced by cell targeted and selective recombinant indicators of neuronal activity. Spontaneous resting state activity is often collected during mesoscale imaging to provide the basis for mapping of connectivity relationships using correlation. However, the information content of mesoscale datasets is vast and is only superficially presented in manuscripts given the need to constrain measurements to a fixed set of frequencies, regions of interest, and other parameters. We describe a new open source tool written in python, termed mesoscale brain explorer (MBE), which provides an interface to process and explore these large datasets. The platform supports automated image processing pipelines with the ability to assess multiple trials and combine data from different animals. The tool provides functions for temporal filtering, averaging, and visualization of functional connectivity relations using time-dependent correlation. Here, we describe the tool and show applications, where previously published datasets were reanalyzed using MBE.

## 1 Introduction

Among the initial goals of the brain initiative was to map the functional activity of potentially every neuron within the human brain.^1^ While this challenge has led to many new approaches to assess connectivity,^2^^–^^–^^6^ it is probably unattainable in the near term. An equally important level of resolution to assess functional relationships is the mesoscale. The mesoscale is an intermediate level of brain functional connectivity between the microscale of cells and synapses and macroscale connections best visualized using whole brain functional magnetic resonance imaging (fMRI) methods.^1^ Through the pioneering work of Grinvald et al., mesoscale connectivity analysis has been well established.^7^^–^^–^^12^ Results from feline and rodent cortex nicely demonstrate the role of large ensembles of neurons that contribute to cortical maps that are shaped by experience and are associated with particular behavioral states.^7^^,^^8^ Complementing the classic strategies of Grinvald and Hildesheim^13^ and Ferezou et al.^14^ are more recent structural connectivity analyses performed by the Allen Institute^15^^,^^16^ and others,^17^^,^^18^ where the projection anatomy of most mouse brain areas can be mapped into a common coordinate framework for C57BL/6 mice.

The level of resolution afforded by mesoscale imaging provides opportunities to compare data across imaging modalities, species, and behaviors.^7^^,^^8^^,^^19^^,^^20^ Fox and Greicius^21^ exploited connectivity relations embedded within spontaneous brain activity in a similar manner to resting-state fMRI. This analysis performed largely within spontaneous events of the cat primary visual cortex, provided a means of assessing functional connectivity relations, which were also present when the animal was given defined visual stimuli.^8^ Recently, our lab and others have taken advantage of large field-of-view (FoV) imaging within the mouse cortex to also assess functional connectivity using spontaneous activity.^12^^,^^22^^,^^23^ This approach, when combined with new structural connectivity information,^15^ indicates that functional connectivity is constrained by major intracortical axonal projections.^12^^,^^24^ This approach of examining relationships within spontaneous events or those stimulated by optogenetics also provides a potential vehicle for broad comparisons between human resting-state fMRI studies and the mouse mesoscale connectome. While these advances, facilitated by the careful insight of Kenet et al.,^8^ have moved the field forward, a significant hurdle exists in processing and interacting with large datasets of mesoscale functional activity. Accordingly, we have built a flexible open-source python tool, which permits significant processing of mesoscale imaging raw data and provides a platform with which others can view and interact with archival datasets (such as widefield mesoscale imaging data from transgenic mice) and explore their own regions of interest, frequencies, or other properties. The tool is further designed so that user-specified plugin pipelines can be created to automate processing steps using existing plugin functions or custom plugins with user-defined additional functions.

One method for inferring functional connectivity from collected spontaneous data would be through the creation of seed pixel correlation (SPC) maps:^25^ a single pixel (or a small region of interest) is selected as the seed. Pearson correlation (zero lag) is used to generate a map showing the extent to which brain activity over time at each pixel correlates with that of the seed.^11^^,^^12^

Correlation matrices are generated from the activity for particular brain regions of interest (RoIs) across relatively long sequences of spontaneous activity. Each RoI–RoI pair consists of two sets of brain activity with a single correlation value for each pair. Pearson correlation coefficients can be computed for each RoI–RoI pair or even all combinations of pixels to generate a connectivity matrix that can be used to infer interareal connectivity relationships.^12^^,^^23^^,^^26^^,^^27^ Using correlation to infer, monitor, and quantify connectivity is common practice in experimental research.^11^^,^^12^^,^^23^ Voxel-based (volume pixel) correlation has been used extensively in human research employing fMRI.^25^ In the case of GCaMP6, this would show us how correlated calcium activity is between selected regions over the time period in which the spontaneous data were collected (typically 3 to 20 min of activity is recorded).

Correlation matrices are forms of functional connectivity analysis. Functional connectivity is defined as the statistical association or dependency among two or more anatomically distinct time-series.^28^ Measures of functional connectivity do not provide information regarding causality or directionality (this is further discussed in Sec. 6). If an analysis of how one region influences another is required, then experimental changes studied via effective connectivity methods are required, which are outside the scope of this paper.^28^

## 2 Materials and Methods

### 2.1 Mesoscale Brain Explorer Executable

Mesoscale brain explorer (MBE) is a cross-platform standalone application (at the time of writing prerelease version 0.7.10 is the latest most stable version available under an MIT license from Ref. 29) that can simply be downloaded and run as an executable without having to set up python or any further dependencies. Moreover, if a python 3.5 environment has been set up and the user has installed all dependencies (see instructions for setting up dependencies in the README: Ref. 30), then the program can be run from the main script via an integrated development environment or the command line. This allows the program to be run on platforms that cannot run executable files. To date, it has been successfully tested on Windows 7, 8.1, 10, and Linux Ubuntu 16.04 systems. Note that a python 2.7 implementation is not provided as python 2.7 will reach its end-of-life in 2020. Python 2.7 users are advised by the Python Software Foundation^31^ to port their code to python 3.5 and we likewise wish to encourage labs to make the switch. A video tutorial that steps through the entire process required to replicate the figures in this paper is provided (see README: Ref. 30). Example image stacks from mouse #0285 used in this manuscript can be downloaded here (see README).

**Fig. 1:**
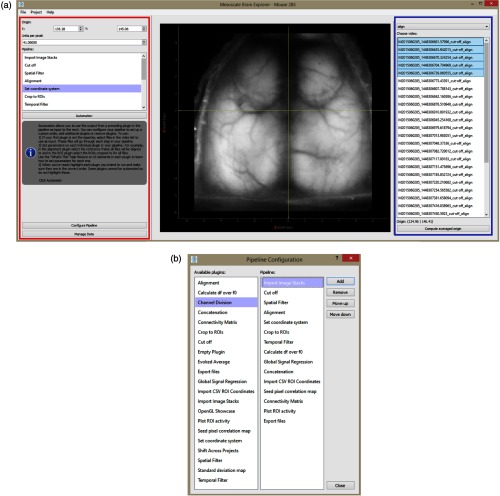
(a) The UI includes the left panel (red) for managing plugins and data common to the project, such as coordinate system origin and pixel width, which here has been set to 41  μm/pixel. The right panel (blue) contains UI elements specific to a selected plugin. Here, we have the “set coordinate system” plugin in view. This plugin is used to set the origin and as the pixel width for the project. Here, we can see that for this project, the five image stacks have been selected. For each one, the anatomical location of bregma was clicked and the origin was taken as the average of all five clicks. (b) Plugins and processing steps used in a particular analysis can be selected and ordered via the Configure Pipeline window.

MBE takes a plugin approach to data processing. Each processing step is independently contained. However, plugins and therefore processing steps used in a particular analysis can be selected, ordered, and saved via the Configure Pipeline window [see Fig. 1(b)].

MBE imports data in the form of stacked .tiffs or .raws, both common file output formats for many imaging systems. Image stacks in our context refer to xy over time. Datatypes uint8, float32 and float64 are supported for .raw file imports, while any datatype is supported for .tiffs as long as its datatype is specified in the file header and supported by numpy.^32^ Multichannel B&W or RGB .tiffs or .raws may be used, however, only a single channel is imported at a time. A user who wishes to use both red and green channels from a single file has to perform the import routine twice. Thereafter, either imported channel data can be operated on in subsequent plugins. All files are converted to python numpy arrays (.npy) upon import and all plugins subsequently assume a .npy format. Any image stack file format is compatible with MBE as long as it can be converted to .tiff, .raw, or .npy format. In a session, all the files imported are contained in a single project.

The user is presented with a graphical user interface (GUI) window, menu and dialog driven interface elements alongside two panels [Fig. 1(a)]. The left panel (red) is used for managing plugins and data common to the project. The right panel (blue) contains user interface (UI) elements specific to a selected plugin.

During analysis, each step is performed with intermediate arrays saved to file. The user can process steps one at a time in any order or set up an automated pipeline, where output of a prior step is taken as an input to process the next step in the pipeline. Pipeline configuration, file paths, the source stack of a processing step, an origin selected for a particular stack, a list of all manipulations a stack has gone through and its type are all saved to a JavaScript Object Notation (JSON) file in the user-defined project directory. Files can be filtered via a dropdown menu [the topmost dropdown menu in the blue region in Fig. 1(a)] based on what manipulations they have gone through making bulk deletion to save disk space easy. Moreover, as long as all data and JSON file are kept together in a single folder with no subfolders, the project can be copied to any supported computer and opened there by MBE with all data and selected processing steps already organized.

MBE is a standalone application and does not assume that the user is familiar with python or the command line. This makes it usable by both programmers and nonprogrammers. Moreover, the source code is structured in a readily extensible framework that can be expanded upon with new plugins developed to suit the specific needs of a researcher (a tutorial on developing your own plugin will be provided in the README). For example, implementing support for different file formats, bandpass filtering techniques, or including additional colormap options for SPC maps (see Appendix A.1.12, A.1.18, and A.1.14) are all possible avenues for further development.

## 3 Experiment

Spontaneous activity collected from an awake female Ai94 mouse^33^ that was previously published was used in this paper’s analysis.^34^ The mouse was head-fixed automatically whenever the mouse entered a chamber to reach its water spout. Brain activity was subsequently imaged through a bilateral transcranial window encompassing the cortex for 30 to 64 s epochs using a [Wave Share Electronics RPi Camera (F)] Raspberry Pi camera at a framerate of 30 Hz with automatic exposure and auto white balance turned off and white balance gains set to unity. A plastic adjustable lens (f=3.6  mm; provided with the camera) was used after unscrewing the lens and placing a 10-mm-diameter green emission filter (ET525/36m, Chroma Technology) between the lens and the imaging sensor. The use of this camera and lens resulted in a bilateral 10.5 to 10.75×10.5 to 10.75 mm FoV and imaging occurred through intact bone.^26^

Sequences of green epifluorescence images using the Raspberry Pi camera are then collected when the mouse is head-fixed. A simple epifluorescence system was used with an LED light source (with excitation 475/30 m and emission filter ET525/36 m Chroma). Collected data (256×256  pixel image stacks) were saved to raw RGB 24-bit files.^34^ A total of 31 such image stacks were recorded.

All the procedures were conducted with approval from the University of British Columbia Animal Care Committee and in accordance with guidelines set forth by the Canadian Council for Animal Care.

## 4 Theory/Calculation

**Fig. 2:**
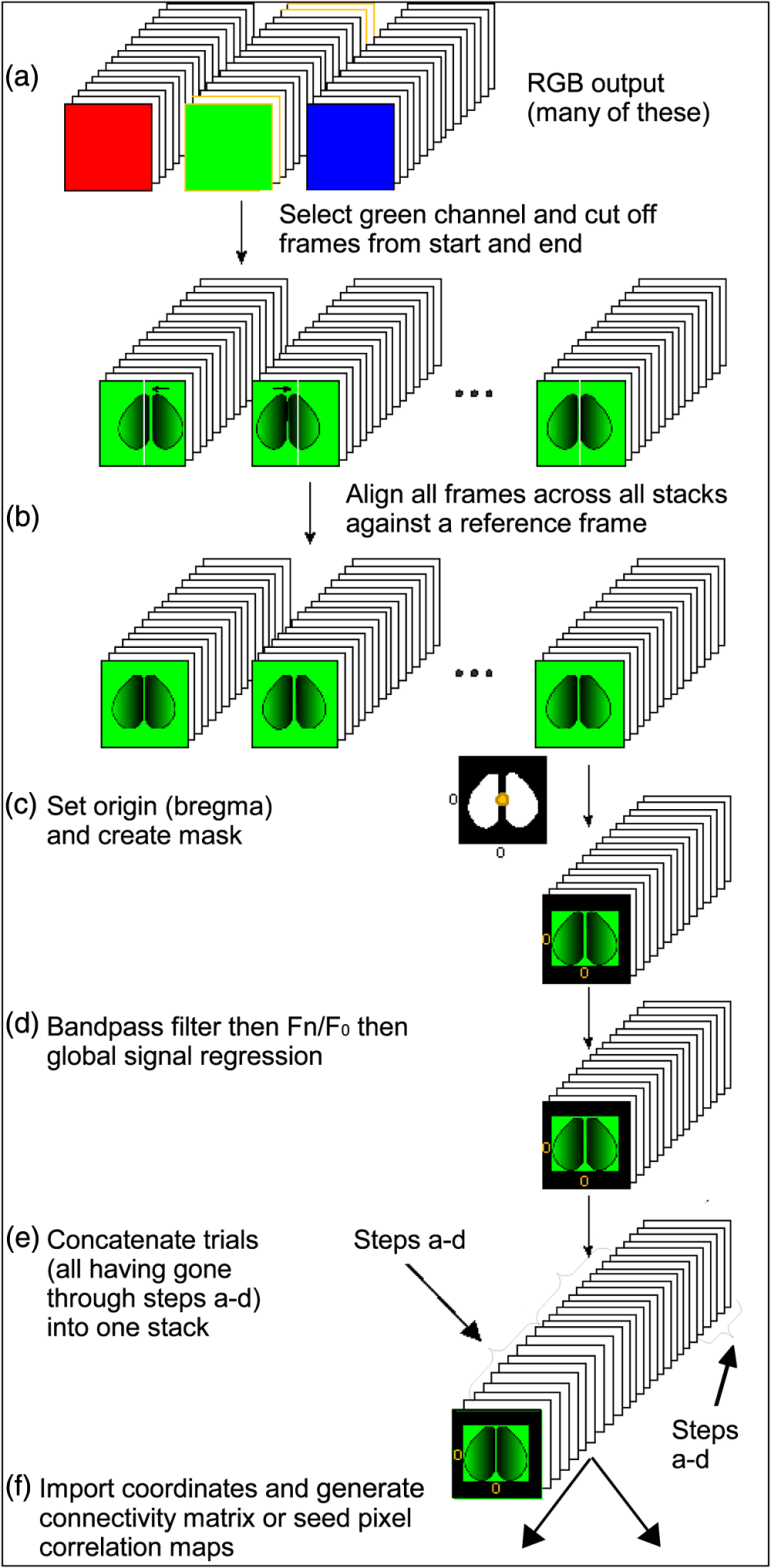
The pipeline we set up specifically for our analysis of mouse #0285. In the application, the ordering of this pipeline can be freely rearranged.

The pipeline we set up specifically for our analysis of mouse #0285 can be visualized in Fig. 2. Note that in the application, the ordering of this pipeline can be freely rearranged. Moreover, many additional available plugins (see Appendix A.1) can also be inserted anywhere in the pipeline. Many of these we do not use in the analysis covered by this paper.

### 4.1 Initial Preprocessing

The second channel (green) from 31256×256  pixel raw image stacks was imported into MBE with no resizing (see Appendix A.1.12).

In our autohead fixing home cage,^34^ mechanical settling of the head fixing mechanism results in movement at the beginning of the recording and we therefore delete the first 20 frames in some image stacks as a precaution.

A single image stack was selected as the template that other stacks were aligned to. All frames in this image stack were sharpened via the unsharp filter plugin (see Appendix A.1.20) using a kernel size of 8. We have previously found^34^ through trial and error that this kernel size sharpens each frame to adequately emphasize the location of blood vessels.

The 400th frame (±13.3  s following trim) to the 500th (±16.6  s following trim) frame of this sharpened image stack were averaged to emphasize the location of the blood vessels further and deemphasize other features, this step is optional and used to fine-tune alignment. Users may opt to simply select a single frame without performing any averaging. This single averaged frame is set as the reference frame. All frames across all image stacks were aligned to this reference frame and aligned to features that were filtered to produce the reference frame—in this case, blood vessels. A fast-Fourier transform was used to translate, rotate, and scale one user-selected frame from each image stack to align it to the reference frame. The translation, rotation, and scale required for this transformation were then applied to all frames in that image stack. This plugin therefore assumes that there was negligible movement within a single image stack (see Appendix A.1.1).

The FoV for the recordings is 10.5 mm meaning each pixel is 41  μm wide [Fig. 1(a)]. The skull anatomical landmark bregma was identified on the first frame of five image stacks via the Set Coordinate System plugin. These five locations were averaged to set the origin globally across all plugins (136.28 pixels, 145.06 pixels; see Appendix A.1.15). This averaging is done to reduce human error that might occur when clicking the location of bregma.

Polygon RoIs were drawn for both left and right hemispheres, masking the cortex border that was imaged as well as most of the brain midline due to the obstructing midline sinus. These are masked as they are sources of non-neuronal noise. In our example, all 31 postalignment image stacks were cropped to the same RoIs (see Appendix A.1.5).

### 4.2 Filtering

A Chebyshev filter (type I digital and analog filter design, order=4, maximum allowable ripple in passband=0.1) with bandpass of 0.3 to 3.0 Hz was applied to all postcropped image stacks (see Appendix A.1.18). This increases the signal-to-noise ratio by removing noise, such as cardiac factors.^9^^,^^11^ Next, the average across all frames was computed to establish a baseline. The change in fluorescence from this averaged baseline for each frame was computed ($\Delta F/F_{0}$). This processing step results in data more robust against slow drifting of the baseline signal and fast oscillatory noise due to tissue pulsation, thus ensuring the signal detected more accurately represents brain activity^35^ (i.e., calcium, glutamate, or voltage transients; see Appendix A.1.2). Although available as an option, no image sharpening (i.e., via an unsharp filter) was performed (see Appendix A.1.20) other than to create a reference frame used in the alignment (see Sec. 4.1).

### 4.3 Global Signal Regression

**Fig. 3:**
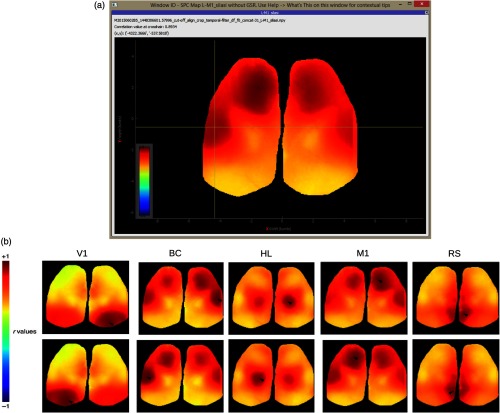
SPC mapping for selected seeds without GSR. (a) MBE UI output of SPC map from the M1 seed in the left hemisphere. The map is of 31 concatenated $\Delta F/F_{0}$ image stacks without GSR applied (i.e., GSR was skipped in the pipeline in Fig. 2). The position of seeds for BC and M1 was adjusted to maximize the remote correlation between them. Their positions are still within the general region of motor and barrel cortex.^36^ The correlation value at the cross-hair (BC) is displayed in the top-left of the window (r=0.8934 with the M1 seed). (b) Correlation maps without GSR applied for seed pixel located in right (upper maps) and left (lower maps) V1, BC, HL, M1, and RS.

**Fig. 4:**
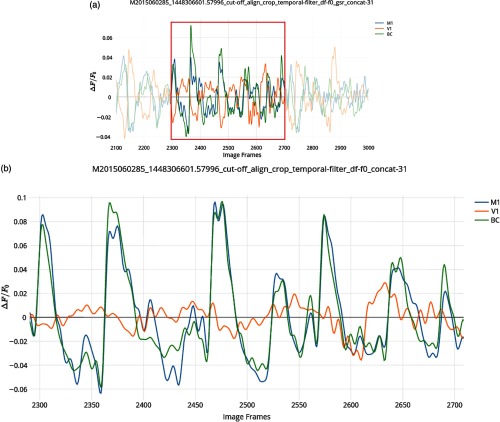
Time plots for selected RoI spontaneous activity without GSR. (a) Zoomed in segment of $\Delta F/F_{0}$ activity without GSR applied between. The 2100th and 3000th frames from all 31 30-s recordings concatenated of spontaneous activity from mouse #0285 (frame rate=30  Hz). $r_{M1-\mathrm{BC}}=0.905\;p\approx 0$, $r_{V1-\mathrm{BC}}=0.0739\;p=0.013$, $r_{M1-V1}=0.144\;p=6.333\times 10^{-6}$. (c) Further zoomed in segment of $\Delta F/F_{0}$ activity between the 2300th and 2700th frames highlighting asynchronous activity between V1 (orange) against BC (green) and M1 (blue). Correlation coefficients for the full 30604 frame time course: $r_{M1-\mathrm{BC}}=0.895$, $r_{V1-\mathrm{BC}}=0.350$, $r_{M1-V1}=0.345$.

Global signal regression (GSR) was applied to all post-$\Delta F/F_{0}$ image stacks, except for Figs. 3 and 4, where GSR was skipped. GSR is a preprocessing technique for removing spontaneous fluctuations common to the whole brain.^37^ GSR involves computing an image stack’s global signal, which is calculated by averaging the signal across all pixels. The global signal is assumed to reflect a combination of resting-state fluctuations, physiological noise (e.g., respiratory and cardiac noise), and other non-neural noise signals. GSR involves a pixel by pixel removal of the global signal by applying a general linear model. GSR has been shown to remove potential global sources of noise, to heighten the contribution of local networks as opposed to brain-wide transitions, thereby facilitating the detection of localized neuronal signals and improving the specificity of functional connectivity analysis.^11^^,^^34^^,^^37^ GSR can also be applied to raw data and this may be advantageous if the image contains areas of variable brightness as low signal areas may be disproportionally weighted.

### 4.4 Concatenation

The entire set of all post-GSR 31 image stacks was concatenated and SPC maps computed for all seeds across the concatenated time series. This is done to use as much spontaneous activity data as possible to improve SPC map accuracy.

### 4.5 Seed Placement

**Fig. 5:**
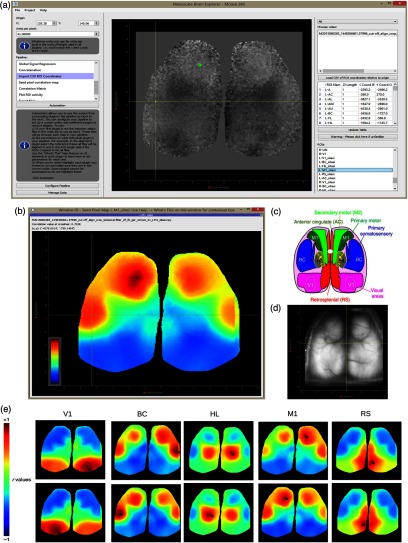
SPC mapping for selected seeds with GSR. (a) UI of the “import CSV RoI coordinates” plugin with M1 seed selected (green RoI) and cross-hair hovering over BC. X and Y coordinates for each seed are loaded from a user-defined CSV that is displayed in the table of the right panel. (b) MBE UI output of SPC Map from the M1 seed in the left hemisphere. The position of seeds for BC and M1 was adjusted to maximize the remote correlation between them. Their positions are still within the general region of motor and barrel cortex.^36^ The correlation value at the cross-hair (BC) is displayed in the top-left of the window (r=0.7028 with the M1 seed). (c) Atlas of the dorsal region of the cortex (adapted from the Allen Mouse Brain Connectivity Atlas.^16^^,^^36^ (d) Raw green fluorescence data from a single frame from an image stack and the location selected for the skull anatomical landmark bregma that is the origin for the coordinate system. (e) Correlation maps for seed pixel located in right (upper maps) and left (lower maps) V1, BC, HL, M1, and RS.

We have previously mapped functional and anatomical coordinates of transgenic mice, confirmed using sensory stimulation in combination with *in vivo* large-scale cortical mapping using channelrhodopsin-2 stimulation.^24^ A csv file was made with coordinates in microns relative to bregma for anterior cingulate (AC), visual cortex (V1), secondary motor cortex (M2), barrel cortex (BC), retrosplenial cortex (RS), primary motor cortex (M1), and the hindlimb cortex (HL) for each hemisphere (see Table 1). Coordinates were added to the project via the Import CSV RoI coordinates plugin displayed in Fig. 5(c) and used as relative distances with respect to bregma (see Appendix A.1.11). This plugin uses the imported coordinates to create square RoIs of user-specified width centered at those coordinates. The size of the RoIs used for SPC mapping was set to 1. SPC maps were thus computed using single-pixel seeds.

**Table 1:** This is a table of the csv file with coordinates in microns the same as those used in Fig. 5(a). This table is also identical to the csv file used to specify the RoIs used in Fig. 7(a). The length column here specifies that all RoIs are square single-pixel wide RoIs. The csv includes RoIs for the anterior cingulate (AC), visual cortex (V1), secondary motor cortex (M2), barrel cortex (BC), retrosplenial cortex (RS), primary motor cortex (M1), and the hindlimb cortex (HL) for each hemisphere (left, L, and right, R). Coordinates were adapted from the Allen Mouse Brain Connectivity Atlas.^16^^,^^36^ The position of seeds for BC and M1 was adjusted to maximize the remote correlation between them. Their positions are still within the general region of motor and barrel cortex.^36^ We previously mapped functional and anatomical coordinates of transgenic mice using sensory stimulation in combination with *in vivo* large-scale cortical mapping using channelrhodopsin-2 stimulation to confirm the coordinates below.^12^^,^^24^

(1) RoI name	(2) Length	(3) X coordinate	(4) Y coordinate
L-V1	1	−2516.8	−4267.8
L-BC	1	−4300	−760
L-HL	1	−1694.2	−1145.7
L-M1	1	−1500	2000
L-M2	1	−870.02	1420.5
L-RS	1	−620.43	−2885.8
L-AC	1	−260	270
R-V1	1	2516.8	−4267.8
R-BC	1	4300	−760
R-HL	1	1694.2	−1145.7
R-M1	1	1500	2000
R-M2	1	870.02	1420.5
R-RS	1	620.43	−2885.8
R-AC	1	260	270

### 4.6 Seed Pixel Correlation Map and Correlation Matrix

SPC maps were generated for all seeds using the concatenated data. A correlation matrix was constructed from single pixel RoIs using the same coordinates and the 31 post-GSR image stack data.

## 5 Results

### 5.1 Seed Pixel Correlation Map Generation

Spontaneous activity was collected during the extended head-fixation of a transgenic mouse expressing GCaMP6 (GCaMP6 mouse), mouse #0285. Correlation maps for seed pixels located in right and left V1, BC, HL, M1, and RS were generated [Fig. 5(e)]. Maps with seeds M1 and BC reveal intrahemispheric synchronous activity between sensory barrel cortex and motor cortices, as previously observed by others.^11^^,^^12^^,^^14^

MBE can output maps to an interactive window [Fig. 5(b)]. Pixel values hovered over by the mouse are displayed at the top of the window. The seed label (X, Y position relative to bregma) can be seen at the end of each window title. Each title additionally contains all processing steps performed. This is useful when outputting numerous plots at the same time from various processing pipelines. All maps are additionally saved as .jpeg files automatically. Here, we can see that the barrel cortex pixel hovered over has r≃0.7 with the M1 seed [Fig. 5(b), top-left]. The user can also click on a pixel to regenerate the map with the selected pixel as the seed.

From the main UI [Fig. 5(a)], the user can see the first frame of the processed image stack with selected coordinates overlaid. The plugin used for seed placement is shown in Fig. 5(a). The user can use the right panel in this plugin to load a csv file that contains micron coordinates. This is displayed in the table in the right panel in Fig. 5(a). Seeds can be selected via the list in the bottom right. Selected seeds are overlaid and displayed on the brain image in the center scene, where RoIs can be reshaped or moved around. Other plugins will likewise have an interactive scene displaying the first frame of the processed image stack between left and right panels [see Fig. 1(a)].

The image of the first frame between the left and right panel in Fig. 5(a) can be clicked on in the SPC plugin (see Appendix A.1.14) to generate an SPC map for the pixel clicked. The user can additionally select any number of image stacks from the first list in the right panel [identical to the list in the right panel of Fig. 5(a)] and any number of seeds from the list in the bottom of the right panel [identical to the list at the bottom in the right panel of Fig. 5(a)] to produce SPC maps in bulk for each seed across all selected image stacks.

**Fig. 6:**
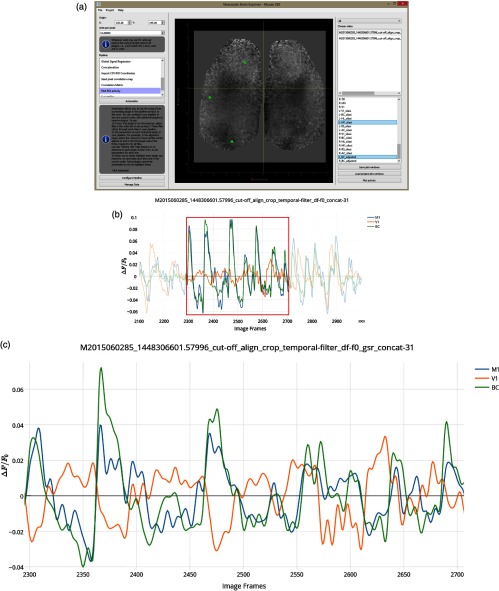
Time plots for selected RoI spontaneous activity with GSR. (a) The main UI with all RoIs to be plotted selected (V1, BC, and M1 in the left hemisphere). (b) Zoomed in segment of $\Delta F/F_{0}$ activity between the 2100th and 3000th frames from all 31 30-s recordings concatenated of spontaneous activity from mouse #0285 (frame rate=30  Hz). $r_{M1-\mathrm{BC}}=0.751p=1.0253\times 10^{-164}$, $r_{V1-\mathrm{BC}}=-0.523p=1.1418\times 10^{-64}$, $r_{M1-V1}=-0.651p=8.229\times 10^{-111}$. (c) Further zoomed in segment of $\Delta F/F_{0}$ activity between the 2300th and 2700th frames highlighting asynchronous activity between V1 (orange) against BC (green) and M1 (blue). Correlation coefficients for the full 30,604 frame time course: $r_{M1-\mathrm{BC}}=0.685$, $r_{V1-\mathrm{BC}}=-0.397$, and $r_{M1-V1}=-0.522$.

In Fig. 6, timeplots of $\Delta F/F_{0}$ activity for selected seeds are shown for mouse #0285 with all 31 image stacks concatenated. Only frames (post cut-off) 2100 to 3000 and 2300 to 2700 are shown. In the application, the output graph is interactive allowing the user to zoom in on the graph to obtain a clear view of the synchrony between M1 and BC (blue and green), while V1 (orange) is poorly synchronized [this is made more clear in Fig. 6(c)]. This is in line with the negative correlation values seen in Fig. 5(e) between V1 and BC or M1.

Pearson correlation coefficients for the full-time course of 30,604 frames are $r_{M1-\mathrm{BC}}=0.685$, $r_{V1-\mathrm{BC}}=-0.397$, $r_{M1-V1}=-0.522$, which agree with the correlation values among these activities in the respective SPC maps [Fig. 5(e)] at these coordinates. All coefficients also agree with r-values previously reported by Silasi et al.:^26^ $r_{M1-\mathrm{BC}}=0.69$, $r_{\mathrm{BC}-V1}=-0.3$, $r_{M1-V1}=-0.53$. Given the large number of samples, all comparisons of BC and M1 activity (with or without GSR) indicated high statistical significance with p-values $<1.0\times 10^{-30}$. We used the barycenter of different regions estimated from Allen Institute anatomical coordinates.^36^ These coordinates do not take into account the possible topography of connections which is why the position of seeds for BC and M1 was adjusted to maximize the remote correlation. Coordinates, however, are still within the general region of motor and barrel cortex.^36^ An advantage of MBE is that the user can open one window for activity plots and another for SPC maps and compare the two to quickly assess the cause of anomalous correlation and adjust coordinates as need be. It is also noteworthy that GSR has been applied to these images to remove global correlations, which tends to make all correlations lower.^25^^,^^37^ To compare this with data, without GSR, see Figs. 3 and 4.

### 5.2 Correlation Matrix

**Fig. 7:**
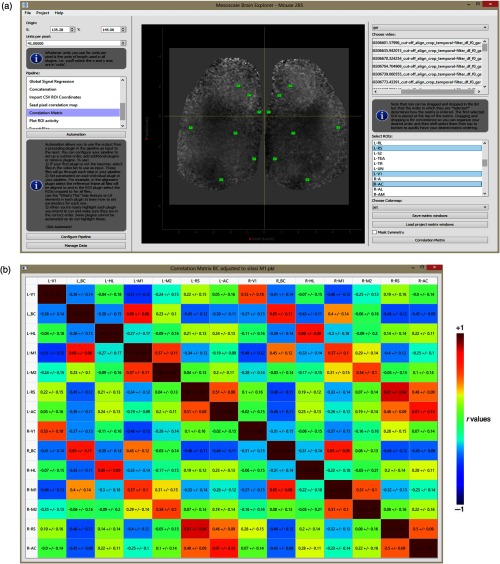
Correlation matrix for selected RoIs with GSR (a) UI of the correlation matrix plugin from where RoIs are selected along with image stacks to generate connectivity matrices. A single image depicting instantaneous $\Delta F/F_{0}$ is shown for RoI placement. (b) Mouse #0285 correlation matrix following collection of spontaneous activity via automated headfix protocols. The data are presented in units of Pearson correlation (r-value) and the stdev reflects variability of r-values between repeated 31 trials.

From the main UI [Fig. 7(a)], the user can see the first frame of the processed image stack (post $\Delta F/F_{0}$) with selected RoIs overlaid. The user can select any number of image stacks from the top right list and any number of RoIs (including custom made RoIs that need not be square) to output a single averaged correlation matrix. Correlation matrices are produced for each selected image stack and selected RoIs, but the final output displays correlation coefficients for a single matrix averaged across all matrices. In this example, we have selected all 31 post-GSR image stacks from mouse #0285. Pearson correlation zero lag (r-value) was computed for each image stack and for each RoI. These values depict how the RoI correlates with other RoIs in the matrix. Standard deviation of r-values for each RoI–RoI pair is computed, showing the variance of the r-value across image stacks [Fig. 7(b)].

## 6 Discussion

For our analysis, we relied on previously collected recordings of spontaneous activity^34^ from awake mice using various fluorescent calcium indicator proteins including GCaMP6.^33^^,^^38^ We present an application for visualizing connectivity relationships in these large datasets that makes them more readily available to the scientific community for analysis (our data is available upon request). A limiting issue with studying spontaneous activity is the sheer amount of data that needs to be collected, stored, and assessed. Our lab has recently developed a system for high-throughput automated head-fixing and mesoscopic functional imaging for transgenic mice within their homecages.^34^ Similar methods were previously developed for rats.^39^ Consequently, a limiting factor in future longitudinal studies will likely be the ease with which collected data can be processed, analyzed, and shared with the community. MBE was designed to ease processing for the end-user by offering a simple interface and application setup. From a design perspective, a plugin approach was chosen for MBE to enhance usability, maintainability, and extensibility: (1) Usability: Different processing steps are clearly separated. The program keeps track of data files and pipeline execution, thus users can focus on their analyses. (2) Maintainability: As each processing step resides in its own plugin, functional units are clearly separated from each other and from the base system. This facilitates the understanding of the software architecture and quick localization of faulty code. (3) Extensible: Processing steps are added as plugins. Plugins are developed independently from this software. They can be inserted without any change to MBE or other plugins and without restarting the application, easing the development of plugins. This framework makes it easier for developers to exchange their own custom-made plugins without having to worry that another developer’s setup may have compatibility issues.

It should be noted that the methods MBE provides are not without their limitations. Perhaps the most pressing limitation is that neither SPC maps nor correlation matrices provide information on connection directionality making causal inference unclear. This may be solved with the addition of plugins that perform Granger causality analysis,^40^ thereby providing diagrams that include nodes for brain regions and arrows denoting the presumed directional flow of brain activity. Alternatively, experimentalists may opt for collecting nonspontaneous activity through techniques, such as channelrhodopsin-2 stimulation, as has previously been undertaken by our lab.^24^^,^^41^^,^^42^ The application supports the use of evoke-triggered data through plugins, such as the evoked average plugin (see Appendix A.1.8).

### 6.1 Temporal Filtering

Temporal filtering spontaneous activity data has its own limitations. Applying a bandpass filter limits our sampling frequency. If the bandpass consists of a sampling frequency range that is too high, fast artifacts such as the mouse’s heart rate are potentially picked up, reducing sensitivity to brain activity. If the bandpass is too slow, artifacts such as hemodynamic processes are accentuated.^27^^,^^43^ Finally, GCaMP6 variants have different decay times following activity, in some cases, limiting the range of frequencies that can be reported.^38^ For studies with corresponding sensory-evoked data, the exact range of a bandpass for spontaneous activity can be selected based on how well the filtered data compares with averaged sensory-evoked data. But for most spontaneous data associated sensory-evoked data is unavailable and therefore, the frequency band is chosen a priori. For transgenic Ai94 GCaMP6 slow mice, we recommend a bandpass filter of 0.3 to 3 Hz with a frame rate of 30 Hz as it shows specificity over green fluorescent protein (GFP).^34^ For Ai93 GCaMP6 fast, a 1 to 10 Hz bandpass was used in a previous study with good specificity over GFP mice that lack functional signals.^26^ Ultimately, this limitation is at least mitigated by MBE in that the interface allows the user to easily modify the filter range and users analyzing sensory-evoked data will not suffer from this limitation (see Appendix A.1.8).

### 6.2 Comparison with Related Software Toolboxes

We here provide an overview of recently developed software toolboxes FluoroSNNAPP, Scintillate, and Vobi One. Vobi One, like MBE, is a software package dedicated to the processing of functional optical imaging data.^44^ It is also written in python and offers a roughly analogous architecture. The GUI likewise has a side-panel from where a user can follow progress or navigate to a particular “process” which, just like a plugin in MBE, is a single script of code running that individual process. The application is likewise extensible, allowing users to add their own custom scripts and add them as “processes” to a custom pipeline. The application makes use of a “condition file” that summarizes info of all imported trials used by the processes and allows for interfacing with external software that cannot directly access BrainVISA (see following paragraph). This is analogous to MBE’s JSON file, which fulfills the same purpose. While MBE provides importing routines for two commonly used versatile file formats .tiff and .raw, Vobi One provides importing routines for two file formats used by two popular CCD camera vendors—.blk files for Optical Imaging Ltd. and .rsd files for SciMedia USA Ltd. Both applications offer spatial binning, however, Vobi One additionally offers temporal binning. As with MBE, upon import files are converted to a single file format that is used across all processes/plugins. Vobi One makes use of NifTI-1, a file format specifically made to foster interoperability at the file-exchange level between fMRI data analysis software packages. MBE, in contrast, simply uses the standard binary file format (npy) offered by the python NumPy package.^32^ Nothing prevents either application from supporting file formats of the other with both offering documentation for supporting additional importing routines.

The main point of departure between the two applications is that Vobi One is integrated with BrainVISA, whereas MBE is not. BrainVISA is an open source software platform that provides a complete modular infrastructure for different neuroimaging software. It organizes heterogeneous software and data, and provides a common general graphical interface across pipelines for different applications. This can essentially provide a view, where each software toolbox comprised of plugins is itself a plugin in BrainVISA. With this integration, Vobi One offers cross-app automation. BrainVISA offers an iterate function allowing the same analysis with steps across toolboxes to be performed on different datasets—i.e., this sets up a loop from the GUI without having to write a program. This automation is much more comprehensive than MBE owing to its integration with BrainVISA. However, MBE does allow the user to string plugins in any order to produce a custom automated pipeline, where all input files are processed through all steps in the pipeline. Instructions for this procedure are provided in the left side panel [Fig. 1(a)]. Vobi One also offers three linear models for denoising optical recordings. The selected model is used to breakdown a recording into its noise and signal components, thereby extracting the fluorescence response.^44^^,^^45^ While Vobi One benefits from BrainVISA integration, MBE is much easier to set up because of its standalone architecture.

Vobi One is, to our knowledge, the only software toolbox with significant architectural and functional similarity to MBE. Two further recently published toolboxes, FluoroSNNAPP and Scintillate,^46^^,^^47^ are related but are aimed at different end-users. FluoroSNNAPP is a MATLAB package for the automated quantification of single-cell dynamics and network activity.^46^ Nothing prevents MBE from being used to generate correlation matrices for cellular recordings, thereby quantifying single-cell dynamics and network activity. Both toolboxes offer $\Delta F/F_{0}$ and RoI drawing functionality. However, FluoroSNNAPP further integrates an automated cell identification method based on spatiotemporal independent component analysis and offers three methodologies for event detection: percentile-based thresholding, wavelet transform decomposing a time-varying signal into frequency and time components, and template-matching using a database of known transient waveforms.^46^ FluoroSNNAPP is thus intended solely for comprehensive microscale analysis.

Scintillate is a MATLAB package that offers real-time $\Delta F/F_{0}$ while image acquisition is on-going, providing the user with signal change information and the means to further refine subsequent acquisitions.^47^ Once signal change has been pinpointed, the user may change objectives, center the image over that specific area, or alter camera settings.^47^ Scintillate is thus intended for use during data collection, while MBE is designed for data analysis after collection and is very appropriate for on the go analysis during an experiment by inexperienced users.

### 6.3 Conclusion

MBE provides a flexible software that is geared first to visualizing connectivity relationships within spontaneous activity data collected using widefield imaging. As a method-agnostic application, MBE is well suited to being used to analyze data from brain activity indicators other than GCaMP, such as voltage-sensitive dye,^12^^,^^27^ glutamate-sensing fluorescent reporter,^48^ or voltage-sensitive fluorescent protein.^9^ The software is also applicable to intrinsic signal imaging^49^ formats and laser speckle imaging, or the flexible architecture can be extended to support any large image dataset. While we have focused on mesoscale functional relationships within a single mouse, the approach could also be used for cellular GCaMP imaging^50^ and the correlation-based tools used to draw functional mapping between individual neurons and their neighbors. MBE also offers a “shift across projects” (see Appendix A.1.16) plugin to align image stacks from different mice onto the same coordinate system, allowing for the generation of connectivity matrices averaged across trials from different mice. A simple division plugin is also included that applies division to selected image stacks. Importantly, dividing fluorescence $\Delta F/F_{0}$ by intrinsic reflectance $\Delta F/F_{0}$ can be used for hemodynamic correction (see Appendix A.1.6).^49^^,^^51^^,^^52^

In conclusion, despite aforementioned limitations in the processing pipeline as well as with interpreting the end result, correlation matrices, SPC maps, and standard deviation maps (see Appendix A.1.17) provide simple and effective methods for identifying patterns of regional mesoscale functional connectivity changes. As an application that standardizes these approaches, that saves each processing step to file and keeps data organized, MBE should be a useful exploratory tool for any person performing functional connectivity analysis.

## Appendix

### A.1 Available Plugins

#### A.1.1 Alignment

This plugin makes use of the python image registration utility imreg_dft to implement a means of optimizing translation, rotation, and scale variation between two images.^53^^,^^54^ The user can decide whether rotation and scale is also accounted for. The user can compute a reference frame that is the x’th to y’th frame (where x,y are user-defined) of a single image stack averaged. A fast-Fourier transform technique is subsequently used to translate, rotate, and scale the z’th frame in each selected image (where z is user-defined) to align this frame to the reference frame. The translation, rotation, and scale required for this transformation is then applied to all frames in that image stack. This plugin therefore assumes that there is negligible movement within a single image stack.

#### A.1.2 Calculate df over f0

This plugin computes the average across all frames to establish a baseline. The change in fluorescence from this averaged baseline for each frame can be computed ($\Delta F/F_{0}$). This processing step results in data more robust against slow drifting of the baseline signal and fast oscillatory noise due to tissue pulsation, thus ensuring the signal detected more accurately represents brain activity^35^ (i.e., calcium, glutamate, or voltage transients).

#### A.1.3 Concatenation

This plugin concatenates selected image stacks into one single stack in the order stacks are selected.

#### A.1.4 Correlation matrix

To generate a correlation matrix, the pixel values in an RoI are averaged for each stack and the resultant one-dimensional array is compared with the arrays from other RoIs. Pearson correlation coefficients are computed for each selected RoI–RoI pair. The averaged correlation value for each RoI–RoI pair across image stacks is outputted in the final matrix. The standard deviation of the correlation values for each RoI–RoI pair is likewise computed and included in the final output. Ultimately, the resultant matrix shows how correlated activity between brain regions is.^27^ Values from the matrix can be saved to a csv file in the project directory.

#### A.1.5 Create regions of interest

MBE was originally inspired by a BMDanalyse, a program designed for the regional analysis of bone mass density through interactive visualizations.^55^ The application makes use of PyQtGraph, a pure python library that leverages numpy for computation and Qts GraphicsView framework for fast display.^32^^,^^56^ It provides well-written classes for RoI-based data slicing on top of the pyqtgraph framework as well as a GUI written in PyQt4, a popular industry-standard framework that supports multiprocessing. All of these tools were adapted from Micheal Hogg’s original program and adapted for use in this plugin for the generation of polygon RoIs and cropping to selected polygon RoIs across a stack of images.

#### A.1.6 Division

Arithmetic division can be performed frame-by-frame between two selected files. An important use case covered by this plugin is potential hemodynamic artifact correction. If diffuse-reflectance isosbestic point signals are used that can reflect blood volume changes, then contamination removal can be performed by dividing the fluorescence $\Delta F/F_{0}$ (typically green epifluorescence) by the reflectance $\Delta F/F_{0}$. Reflectance $\Delta F/F_{0}$ is typically sourced from green light or, in some cases, a blue reflection image also near an isobestic point, given intrinsic hemodynamic blood volume signals can be measured with either.^49^^,^^51^^,^^57^

#### A.1.7 Empty plugin

This is a template plugin that developers can use. It provides a list of image stacks that can be selected to provide an interactable pyqtgraph view of the first frame of the selected image stack along with an x-y coordinate frame, a drop-down list for filtering image stacks according to the last processing step it went through as well as a video player to view all frames in an image stack with a slider once a list item is double-clicked. Finally, a button provided is connected with an empty function that a developer could expand.

#### A.1.8 Evoked average

The response to sensory stimulation (light flashes) can be recorded and averaged to map visual cortical areas.^11^ These sensory evoked averages are referred to as motifs.^12^ The propensity for spatial–temporal activity motifs to repeat in a set of spontaneous activity can then be assessed.^12^

This plugin averages selected image stacks across image stacks to create an averaged image stack. This operation is typically used to generate the aforementioned sensory or behavior evoked average motifs.

#### A.1.9 Export files

Image stacks can be exported to .tif, .raw, or .mp4 formats. Further file format support could be implemented within this plugin.

#### A.1.10 Global signal regression

GSR can optionally be applied to remove spontaneous fluctuations common to the whole brain using a general linear model. GSR has been shown to facilitate the detection of localized neuronal signals and improve the specificity of functional connectivity analysis.^37^

#### A.1.11 Import csv regions of interest coordinates

Please refer to the README for instructions regarding how to structure coordinates to be used by this plugin (Ref. 30).

Square RoIs are drawn at brain locations using coordinates specified by the user via a .csv or .txt file, which is loaded. Each RoI additionally has its own custom size. All RoIs loaded are saved to the project directory as a .RoI file to be used across plugins.

#### A.1.12 Import image stacks

.tif and .raw are fully supported with .tiff reading handled by tifffile.^58^ All files are converted to python numpy arrays (.npy) upon import. .npy formats can also be imported directly. Support for other formats can be implemented within this plugin.

#### A.1.13 Plot regions of interest activity

RoI activity across stacks can be plotted for all selected RoIs. This plugin opens an interactive pyqtgraph GraphicsWindow, where the graph of activity can be manipulated (e.g., zoomed in on) before being exported to an image file, scalable vector graphic, matplotlib window, csv, or HDF5^56^ [Fig. 6(b)] shows data that has been exported from the plugin to a csv. The graph was subsequently made using Plotly,^59^ an online data visualization and analytics tool.^56^

#### A.1.14 Seed pixel correlation map

The user can click a single pixel called the seed. Pearson correlation zero lag is used to generate a color map showing how brain activity over time at each pixel correlates with brain activity at the seed.^11^ SPC maps thus reveal brain regions displaying synchronous activity.

The user can also use a list of defined seeds with defined locations from the .csv or .txt file loaded via the import CSV RoI coordinates plugin. This will either generate SPC maps in a separate window for each selected seed or simply save SPC maps for all seeds across all selected image stacks to the project directory as .jpeg files and as .npy files that store all pixel values.

#### A.1.15 Set coordinate system

The origin, used as a reference point for user-specified coordinates, can be specified at this plugin. The user can select an origin per image stack. This value is stored for each individual files JSON parameter. An averaged origin across selected file origins can then be generated to set the origin for the entire project. In the typical use-case, X and Y are centered on the anatomical landmark bregma.

#### A.1.16 Shift across projects

MBE works with a single project, wherein a JSON file defines all plugins and data in the project directory used by MBE for that project. A single project has a single origin across all files. In our example, the origin was set to be the location of bregma. As such, using image stacks across different mice is not feasible as the algorithm used by the alignment plugin is strongly influenced by blood vessels. It is much more feasible to shift all stacks from other animals to match the origin of the current project. This is what this plugin achieves, allowing the user to shift and then immediately import specific selected image stacks from multiple projects. This is needed, for example, in the generation of averaged correlation matrices across many image stacks from different mice.

#### A.1.17 Standard deviation map

The user selects a maximum value for standard deviation and the plugin computes a standard deviation map showing how much brain activity varies over time at each pixel. The max value is taken as the upper limit of the map scale. The user can also specify a maximum standard deviation, limiting the upper bound of the scale.

#### A.1.18 Temporal filter

A temporal filter can be applied to all selected image stacks, with the user specifying the passband of allowed brain activity signal. This increases the signal-to-noise ratio by removing noise, such as cardiac factors.^9^^,^^11^ Currently, the only filtering algorithms MBE provides is Chebyshev (type I digital and analog filter design, order=4, maximum allowable ripple in passband=0.1) with high and low passbands and frame rate user-specified. This linear MATLAB-style infinite impulse response filter is subsequently applied once forward and once backward across stacks for each selected stack.^60^ Other filtering algorithms, such as butterworth—also available from the same python package as the Chebyshev filter—could easily be implemented into this plugin.

#### A.1.19 Trimming

The user can define how many frames can be discarded from the start and end of all selected image stacks. This plugin is primarily for cleaning the initial data (e.g., removing movement artifacts at the start and end of a recording).

#### A.1.20 Unsharp filter

An unsharp filter subtracts an “unsharp,” or smoothed, version of an image from the original image. This outputs an image with enhanced edges.^61^

Each frame in the image stack is first smoothed. The size of the mean filter kernel is selected and each frame in the selected image stacks is convolved with the given kernel. This smooths each frame by reducing the variation between one pixel and the next with kernel size controlling the magnitude of smoothing. This filtered image stack is subtracted from the original image stack frame by frame, thereby prominently highlighting (i.e., sharpening) features in the stack that are a particular size, relative to the kernel size.

Unsharp filtering is often used to highlight blood vessels. A frame from the sharpened stack is then used as the reference frame for alignment (see Appendix A.1.1) such that frames across other image stacks are aligned based on the location of blood vessels in the reference frame.
